# RETRACTION: P-Glycoprotein Exacerbates Brain Injury Following Experimental Cerebral Ischemia by Promoting Proinflammatory Microglia Activation

**DOI:** 10.1155/omcl/9837687

**Published:** 2025-11-07

**Authors:** Oxidative Medicine and Cellular Longevity

RETRACTION: Y. Chen, X. Fei, G. Liu, et al., “P-Glycoprotein Exacerbates Brain Injury Following Experimental Cerebral Ischemia by Promoting Proinflammatory Microglia Activation,” *Oxidative Medicine and Cellular Longevity* 2023, no. 1 (2023): 1–18, https://doi.org/10.1155/2023/6916819.

The above article, published online on 16 December 2023 in Wiley Online Library (wileyonlinelibrary.com), has been retracted by agreement between the journal Editor-in-Chief, Jeannette Vasquez Vivar; and John Wiley & Sons Ltd. The retraction has been agreed following an investigation into image duplication raised by the authors and third parties, including comments on PubPeer [1], as follows:

• Figure 3a: Column 1 row 4 (NCsiRNA - CD206/Iba-1) and column 3 row 2 (NCp-AAV - CD16/Iba-1). Small sections of overlap, suggesting both originate from the same image.
• Figure 7a: Column 2 row 2 (NCsiRNA - iNOS/DAPI) and column 4 row 4 (NCpcDNA3.1 - Arg-1/DAPI). Whole image duplication.
• Figure 7a: Column 2 row 5 (P-gppcDNA3.1 - iNOS/DAPI) and column 4 row 5 (P-gppcDNA3.1 - Arg-1/DAPI). Whole image duplication.
• Figure 7a and 8a: Figure 7a column 1 row 2 (NCsiRNA - CD16/DAPI) and Figure 8a column 1 (GR/DAPI – Control). Whole image duplication.

Additional image duplication was subsequently identified in Figure 1a and 1b concerning the western blot bands for β-Actin, which had been rotated 180° and brightness altered.

The authors' explanations and proposed corrections did not sufficiently address the publisher's concerns. In light of the multiple image issues, the data, and the conclusions are considered unreliable. The authors were informed of the decision and disagreed with the retraction.
